# Production and Characterization of Biocomposite Films of Bacterial Cellulose from Kombucha and Coated with Chitosan

**DOI:** 10.3390/polym14173632

**Published:** 2022-09-02

**Authors:** Julia M. Márquez-Reyes, Rubí E. Rodríguez-Quiroz, Juan P. Hernández-Rodríguez, Beatriz A. Rodríguez-Romero, Héctor Flores-Breceda, Juan Napoles-Armenta, Itzel C. Romero-Soto, Sergio A. Galindo-Rodríguez, Juan G. Báez-González, Mayra Z. Treviño-Garza

**Affiliations:** 1 Facultad de Agronomía, Universidad Autónoma de Nuevo León (UANL), Francisco I. Madero S/N, Ex Hacienda el Cañada, Escobedo 66050, Mexico; 2 Centro Universitario del Norte, Universidad de Guadalajara, Km. 191, México 45D No. 23, Colotlán 46200, Mexico; 3 Facultad de Ciencias Biológicas, Universidad Autónoma de Nuevo León (UANL), Av. Pedro de Alba S/N, Cd. Universitaria, San Nicolás de los Garza 66455, Mexico

**Keywords:** green tea, kombucha, bacterial cellulose, chitosan, coatings

## Abstract

The purpose of this research is to produce and characterize bacterial cellulose (BC) films coated with chitosan (BC-CH). BC films were produced in a fermentation medium based on *Camellia sinensis* tea and dextrose (12 days at 25 °C) and subsequently treated with coating-forming solutions (CFSs) based on chitosan (BC-CH 0.5%, BC-CH 1.0%, and BC-CH 1.5%). As a result, the FTIR spectra of BC and BC-CH 1.5% showed the main characteristic bands of cellulose and chitosan. In the physicochemical characterization of the films, it was found that the incorporation of the chitosan coatings did not affect the thickness; however, it decreased the luminosity (L*) and increased redness (a*), yellowness (b*), and opacity (75.24%). Additionally, the light absorption properties in the UV-Vis range were improved. Furthermore, the application of the CFSs increased: the solubility (64.91%), the antimicrobial activity against *S. aureus* (6.55 mm) and *E. coli* (8.25 mm), as well as the antioxidant activity (57.71% and 24.57% free radical scavenging activity), and the content of total phenols (2.45 mg GAE/g). Finally, our results suggest that the BC-CH films developed in the present study show a potential application as active packaging material for food.

## 1. Introduction

Tea is one of the most popular beverages in the world and ranks second in consumption after water as it is a non-alcoholic beverage with caffeine. It is obtained by infusion processes of different plant species, one of the main ones being *Camellia sinensis*. [1]. The use and application of bioactive compounds from green tea in the food industry are promising, due to its high content of antioxidants that can act in different ways on foods through molecular interaction/modification mechanisms, allowing the development of innovative, healthy, nutritious, and long shelf-life products [2,3].

Moreover, cellulose (β (1→4) linked D-glucose unit polysaccharide) is the most abundant biological molecule on the planet; it is a biopolymer that is insoluble in water due to its linear structure [4]. It presents hydrogen bridges between the hydroxyl groups of different chains linked to each other with the glucose that make it impenetrable to water [5]. Marine algae and prokaryotic microorganisms produce cellulose without lignin and hemicellulose with high purity, significant water retention capacity, high mechanical strength and porosity, biocompatibility, and high crystallinity [6]. Due to its characteristics, this bacterial cellulose (BC) has excellent opportunities to be used for different industrial applications in food, medical, environmental, and textiles, among others [7,8,9]. According to their different forms of production, specific macrostructural characteristics are generated [10], which can be modified after production to improve their chemical, biological, and mechanical properties.

Furthermore, in food engineering, biopolymers have been used to coat foodstuffs, leaving behind synthetic materials, and contaminating compounds [11]. Chitosan (N-acetyl glucosamine and D-glucosamine linked by β (1→4) glycosidic bonds) is a widely distributed polymer; the interest in this polymer as a base for edible films/coatings is due to its bactericidal and fungicidal activity, and other properties such as being biodegradable and low-cost [12]. Additionally, chitosan combined with BC has been used in dye removal, drug immobilization, antioxidant agents, and wound dressings, among other applications [13].

On the other hand, in the search to provide more new alternatives, as well as to develop more efficient materials or with improved properties, several studies have focused on the production of: cellulose-based films [14,15,16], bilayer films based on chitosan/cellulose nanocrystals [17], bilayer-structured regenerated cellulose/chitosan films [18], chitosan/kombucha-tea active films [19], chitosan/tea polyphenols active films [20], multilayer coated-chitosan paper [21], cellulose acetate, and regenerated cellulose films [22], among others, for various applications. Therefore, this research aims to produce and characterize bacterial cellulose (BC) and chitosan-coated BC films (BC-CH).

## 2. Materials and Methods

### 2.1. Production Process of BC Films

First prepared was the culture medium based on *Camellia sinensis* (Hill Country Fare), where 1 L of distilled water at 85 °C was used, to which 3.6 g of green tea and 55 g of dextrose were added. The mixture was allowed to stand for 15 min, filtered, and cooled to room temperature (25 ± 2 °C). Then, in cylindrical plastic containers (500 mL capacity), 200 mL of the fermentation medium was added and inoculated with the microorganism *Acetobacter* sp. (5% *v*/*v*). The inoculum was provided by the Microbial Biotechnology Laboratory of the Faculty of Agronomy, UANL. Next, the containers were covered with gauze and left to ferment for 12 days at 25 ± 2 °C (Heratherm^TM^ Incubator, Thermo ScientificTM, Waltham, MA, USA) in complete darkness. Once the fermentation time had elapsed, the BC discs were removed from the culture medium by eliminating excess water.

#### Pretreatment and Performance of BC Films

The BC disks obtained in the previous stage were subjected to an immersion process in an NaOH solution (1 N) followed by an immersion process in distilled water for 24-h periods. Finally, the BC discs were recovered and dried at 55 °C in a forced convection oven (Felisa, Zapopan, Jal, Mexico). Additionally, the yield of BC films was calculated by considering the initial fresh weight of the disc before pretreatment and the dry weight of the disc after pretreatment [9].

### 2.2. Development of Chitosan-Coated BC Films

Firstly, three coating-forming solutions (CFSs) based on chitosan (0.5, 1.0, and 1.5%) containing acetic acid (0.5%) and glycerol (0.5%) were developed. Chitosan of 310,000–375,000 Da (molecular weight) with a degree of deacetylation of 75–85% (Sigma Aldrich, Saint Louis, MO, USA) was used. The CFSs were prepared in distilled water (100 mL) by constant mechanical stirring for 24 h until a homogeneous solution was obtained. The concentrations of the components in the CFSs were established based on previous experiments (data not shown). To obtain chitosan-coated BC films (BC-CH 0.5%, BC-CH 1.0%, and BC-CH 1.5%), the BC films were immersed in the different CFSs for 30 min. BC films without any treatment were used as controls. After immersion, the films obtained were removed from the CFSs and placed in a convection oven (Felisa, Zapopan, Jal, Mexico) at 55 °C until a constant weight was obtained.

### 2.3. Rheological Characterization of CFSs

The rheological characterization of each CFS was performed using a ReolabQC rotational rheometer (Anton Paar, Graz, Australia) using CC-39 geometry at a shear rate of 1–100 s^−1^ (inverse seconds) and a temperature of 25 °C. Shear stress and viscosity were expressed as Pascal (Pa) and millipascal-seconds (mPa·s), respectively.

### 2.4. Characterization of BC and BC-CH Films

#### 2.4.1. Fourier Transform Infrared Spectroscopy (FTIR)

The BC and BC-CH 1.5% films obtained in this study were selected to be characterized by FTIR in combination with the attenuated total reflectance (ATR) technique on a PerkinElmer Spectrum (version 10.4.00; UATR, Perkin Elmer, Waltham, MA, USA) in the range of 4000 to 400 cm^−1^ (inverse centimeters), in absorbance units (a.u.) with an ATR accessory at 4 cm^−1^ resolution, in 64 scans with baseline adjustment.

#### 2.4.2. Thickness Measurements

The thickness of the BC and BC-CH films was measured using a digital micrometer (Her-411, Steren, Azcapotzalco, CDMX, Mexico), and each disk was measured at six different points covering its entire perimeter [14]. Results were expressed as average thickness (millimeters; mm) for each treatment (*n* = 4).

#### 2.4.3. Color Analysis

A color analysis of the BC and BC-CH films (*n* = 4) was performed using a portable colorimeter (Serie SP60, X-Rite, Asia Pacific Limited, Hong Kong, China). Results were presented on a five-stimulus scale using the CIELAB color parameters, L* (lightness, 0 black to 100 white), a* (−green to +red), and b* (−blue to +yellow) [23].

#### 2.4.4. Opacity Analysis

Measurements were performed at four different points on the BC and BC-CH films (*n* = 4) using a colorimeter (Serie SP60, X-Rite, Asia Pacific Limited, Hong Kong, Chinese), as reported by Fakhouri et al. [24]. First, the opacity values were calculated using the following equation: % Opacity = (Opacity of the film on a black background/Opacity of the film on white background) × 100.

#### 2.4.5. Ultraviolet-Visible Spectroscopy (UV-Vis)

The barrier properties of the BC and BC-CH films (*n* = 4) against UV and visible light were estimated according to Haghighi et al. [25], with some modifications. First, a UV-Vis spectrophotometer (Evolution™ 201/220 UV-Vis Thermo Scientific™, Waltham, MA, USA) was used, and a scan from 300 to 800 nm was performed using rectangular film (1 × 4 cm^2^). Results were expressed in absorbance units (a.u.).

#### 2.4.6. Water Solubility Tests

The methodology proposed by Wang et al. [20] was used, with some modifications. Rectangular films (1 × 4 cm^2^; *n* = 4) were used, and their initial weight was determined by drying them at a temperature of 55 °C for 24 h. Subsequently, they were placed in conical tubes with 15 mL of distilled water and kept in agitation at 100 rpm for 24 h. After the agitation time had elapsed, the final weight of the films was determined under the conditions mentioned above. Water solubility was determined using the following equation: % Solubility = (Initial dry weight − Final dry weight/Initial dry weight) × 100.

### 2.5. Biological Activity Determinations 

#### 2.5.1. Antimicrobial Activity Tests

In these assays, *Staphylococcus aureus* bacteria were used as Gram (+) and *Escherichia coli* as Gram (−). The strains were cultured in 10 mL of brain heart infusion broth at 37 °C for 16 h. After the incubation period, the cell concentration of each microorganism was adjusted to 1 × 10^8^ cells/mL according to the McFarland scale in a spectrophotometer (Evolution™ 201/220 UV-Vis Thermo Scientific™, Waltham, MA, USA) at 625 nm, according to the methodology reported by Treviño-Garza et al. [26]. Antimicrobial activity tests were performed by the agar diffusion method using Petri dishes containing brain heart infusion agar (20 mL) and inoculated (0.1 mL) with each microorganism. To have homogeneous diameters (7 mm), the discs of the BC and BC-CH films (*n* = 4) were obtained with the help of a sterile circular perforator. Subsequently, these discs were placed on the agar surface of the Petri dishes previously inoculated and incubated (Incubator, Herather™, Thermo Scientific™, Waltham, MA, USA) at 37 °C for 24 h. Finally, antimicrobial activity was determined by measuring the inhibition halo (mm) [26].

#### 2.5.2. Antioxidant Activity Tests

##### Obtaining Alcoholic Extracts

For all determinations, the same procedure of obtaining the methanolic extract from the films was performed according to the methodology reported by Ashrafi et al. [19], with some modifications. First, the BC and BC-CH films (0.5 g) were macerated in 10 mL of methanol and allowed to stand for 24 h in complete darkness. As described below, corresponding volumes were then taken to determine the total phenolic content and antioxidant activity.

##### Total Phenolic Content

The total phenolic content of all developed films (*n* = 3) was determined by the Folin-Ciocalteu procedure [27] and expressed as mg gallic acid equivalents (GAE)/g of dry weight. The concentration was calculated independently in triplicates (*n* = 3). The determination was carried out with 250 µL of the methanolic extract, 800 µL of water, 50 µL of Folin-Ciocalteu reagent (1 N), and 800 µL of Na_2_CO_3_ at 7.5%. Subsequently, they were kept for 30 min in the dark, and the reading was performed in a spectrophotometer (Evolution™ 201/220 UV-Vis Thermo Scientific™, Waltham, MA, USA) at 750 nm.

##### DPPH Radical Scavenging Activity

The antioxidant activity of BC and BC-CH films (*n* = 3) was evaluated using tests for scavenging of free radicals of DPPH (2,2-diphenyl-1-picrylhydrazyl) following the methods of Blois [28], Siripatrawan, and Harte [29], and Wang et al. [20], with some modifications. From the methanolic extract, 0.050 mL was taken and mixed with 1 mL of the DPPH solution. The mixture was then incubated at room temperature for 30 min, and the absorbance at 517 nm was determined using a spectrophotometer (Evolution™ 201/220 UV-Vis Thermo Scientific™, Waltham, MA, USA) [30]. Changes in the absorbance were measured, and antioxidant activity was expressed as % DPPH radical scavenging activity using the following equation: DPPH radical scavenging activity (%) = (absorbance of DPPH solution − absorbance of sample/absorbance of DPPH solution) × 100 [19,20].

##### ABTS Radical Scavenging Activity

The antioxidant activity of BC and BC-CH films (*n* = 3) was determined by measuring the free radical scavenging activity using the ABTS (2,2’-azino-bis (3-ethylbenzothiazoline-6-sulfonic acid)) method [31,32]. For analysis, an aqueous solution of ABTS (7 mM) and potassium persulfate (140 mM) was prepared and kept in the dark for a period of 16 h; subsequently, the absorbance of the solution was measured at 734 nm using a spectrophotometer (Evolution™ 201/220 UV-Vis Thermo Scientific™, Waltham, Ma, USA). Later, the solution was diluted with distilled water to obtain an absorbance value between 0.7 and 1.00 at this wavelength. Next, the 25 µL of the methanolic extract was mixed with 1 mL of ABTS reagent and kept in the dark for 45 min. Changes in the absorbance were measured, and antioxidant activity was expressed as % ABTS radical scavenging activity using the following equation: ABTS radical scavenging activity (%) = (absorbance of ABTS solution − absorbance of sample/absorbance of ABTS solution) × 100 [32].

### 2.6. Statistical Analysis

The data obtained from the different analyses were subjected to an analysis of variance (ANOVA) followed by multiple comparisons of means tests (Tukey test), using a significance level of 0.05. These analyses were performed using SPSS software (IBM version 25, SPSS Inc., Chicago, IL, USA).

## 3. Results and Discussion

### 3.1. BC Films Yield

**Figure 1:**
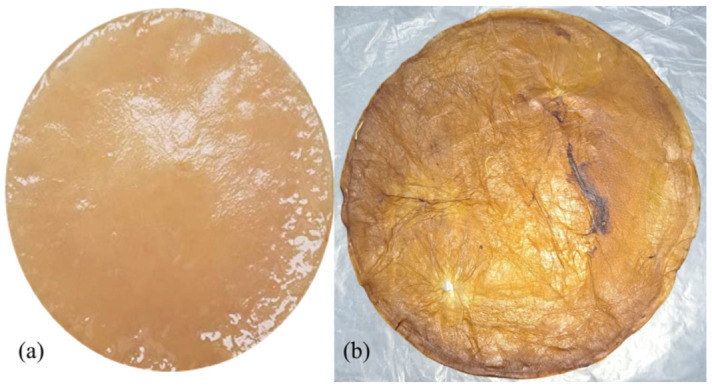
Appearance of: (**a**) Wet BC, and (**b**) dry BC films produced in a culture media based on green tea after 12 days of fermentation at 25 °C.

The yield of BC films (dry basis; Figure 1) derived from kombucha was 8.3 ± 2.6 g/L; similar results were reported by Treviño-Garza et al. [14] in microbial cellulose films produced in a culture medium based on *Camellia sinensis* tea and sucrose.

### 3.2. Rheological Analysis of CFS

**Figure 2:**
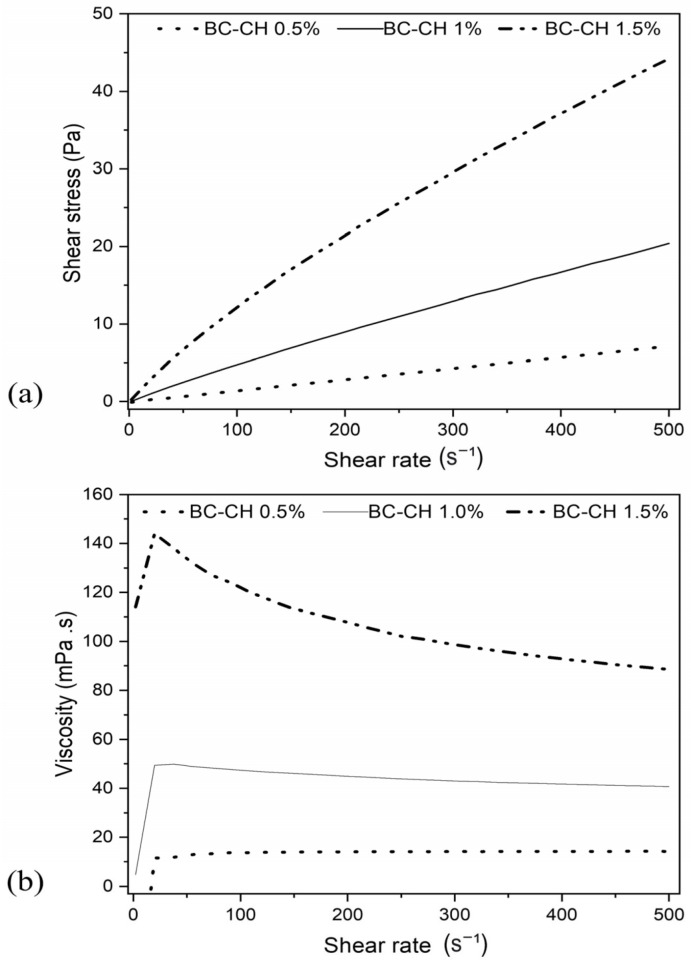
(**a**) Flow curve, and (**b**) viscosity curve of CFS prepared with different concentrations of chitosan.

Regarding the rheological analysis of the CFSs, in the flow curves, it was found that CH 0.5%, CH 1.0%, and CH 1.5% solutions showed the behavior of Newtonian fluids since a linear relationship exists between the shear stress and shear rate. Figure 2a shows that as the concentration of chitosan increases, the slope of the curve increases. Likewise, in the viscosity curves (Figure 2b), this parameter remains constant regardless of the shear stress or shear rate. Finally, the highest viscosity was presented by the formulation CH 1.5% (107 ± 15 mPa.s), followed by CH 1.0% (43 ± 3 mPa.s), and CH 0.5% (14 ± 1 mPa.s). The findings agree with those found in previous studies [33,34], where a Newtonian fluid behavior has been reported at low concentrations of chitosan (0.2–1.0%), with a tendency towards non-Newtonian pseudoplastic behavior as the polymer concentration increases (0.5–3.0%). This behavior can be attributed to a restriction in the freedom of movement in the polymeric chains and an increase in the degree of entanglement when increasing chitosan concentrations [33].

### 3.3. Obtaining BC-CH Films

The BC and BC-CH films obtained in this investigation (Figure 1) showed a brown color, a relatively homogeneous appearance, a thick appearance, and little flexibility, similar to the reports in previous studies [14].

### 3.4. Physicochemical Characterization of BC and BC-CH Films

#### 3.4.1. Fourier Transform Infrared Spectroscopy (FTIR)

**Figure 3:**
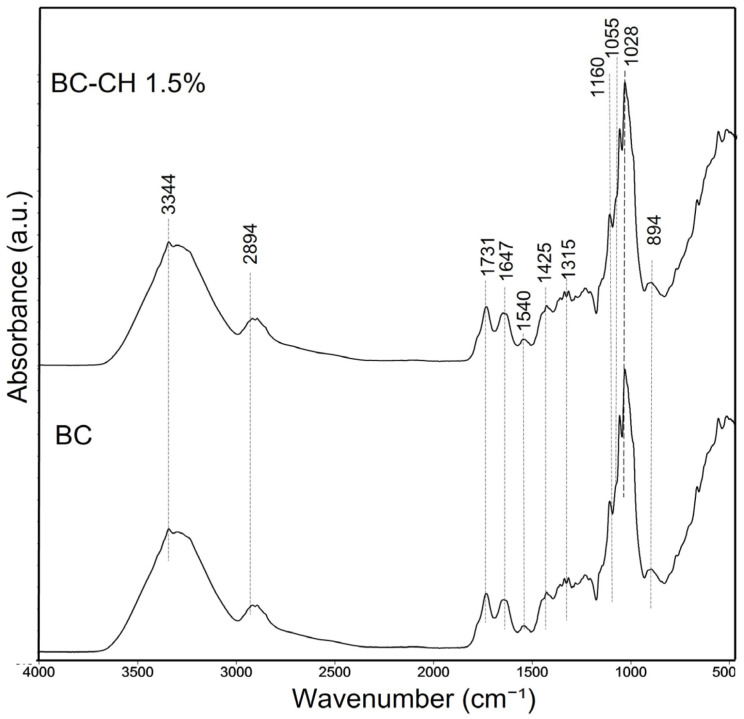
FTIR spectra of BC and BC-CH 1.5% films.

The FTIR spectra showed similar signals in the frequency and intensity of the peaks in BC and BC-CH 1.5% films. In Figure 3, a broad peak was observed at 3343 y 3344 cm^−1^, corresponding to the stretching vibration of the hydroxyl groups (OH) involved in inter- and intramolecular hydrogen bonds [16]. On the other hand, the absorption band at 2894 cm^−1^ is related to the C–H asymmetric stretching of aliphatic CH_2_ and CH_3_ groups [35]. Furthermore, the signals found in the 1731–1733 cm^−1^ and 1647 cm^−1^ regions are related to the C=O stretching [36,37]. The intensity of the signals increased with chitosan. The signals of 1428–1425 cm^−1^ and 1315 cm^−1^ are attributed to the CH_2_ symmetric stretching and out-of-plane wagging of the CH_2_ groups, respectively [38]. The absorbance band 1428 cm^−1^ is attributed to the highly ordered (crystalline) regions in bacterial cellulose [39]. In addition, the absorption bands found at 1540 cm^−1^ are related to the vibrations of chitosan’s protonated amino group (NH_3_^+^) [20]. Although a similar signal is observed in BC (1535 cm^−1^), this effect could be associated with the presence of amino acids in the polymeric matrix of BC produced during the kombucha fermentation process [19]. Meanwhile, the peaks found in the range 1104–1106 cm^−1^ can be associated with C–O–C vibrations, while the peaks 1025–1051 and 1028–1055 cm^−1^ are related to C–O, and C–O–H vibrations, respectively [40]. Finally, the absorption peak at 894 cm^−1^, known as the “amorphous band,” is related to the out-of-phase ring stretching of the -1,4-glycosidic bonds in bacterial cellulose [38]. The fingerprint regions found in both FTIR spectra are characteristics of cellulose and chitosan.

#### 3.4.2. Thickness

**Figure 4:**
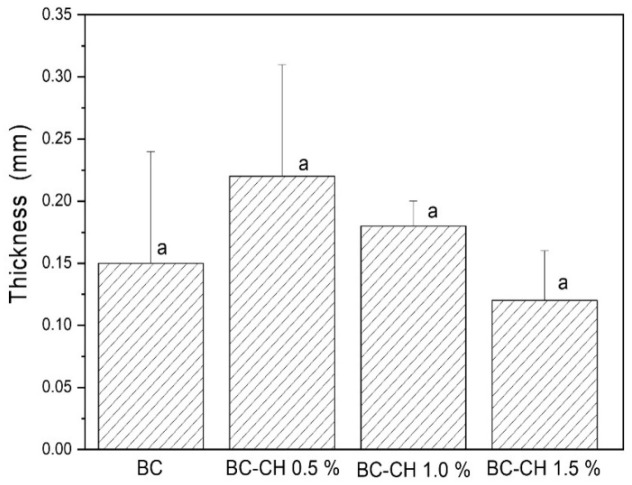
Thickness of BC and BC-CH films coated with various concentrations of chitosan. Average ± standard deviation. Letter (a) indicates no significant differences (*p* > 0.05) between treatments.

According to what was reported by Betlej et al. [15], the thickness of BC films produced by kombucha microorganisms is related to the type and amount of nutrients in the fermentation medium (carbon sources, nitrogen, and other additives such as the type of tea) and can fluctuate between 0.04–0.46 mm. On the other hand, according to previous studies on bilayer films based on chitosan/cellulose nanocrystals, an increase in the thickness of the films was reported as the number of layers in the formulation increased [17]. According to our current research (Figure 4), no significant effect (*p* < 0.05) was found in the chitosan concentration on the thickness of the BC-CH films, whose values were 0.15 ± 0.11 (BC), 0.22 ± 0.09 (BC-CH 0.5%), 0.18 ± 0.02 (BC-CH 1.0%), and 0.12 ± 0.03 (BC-CH 1.5%); this is in agreement with the values reported by Betlej et al. [15]. Additionally, the thickness values of our films were like those reported in bilayer-structured regenerated cellulose/chitosan films [18].

#### 3.4.3. Color Properties and Opacity

**Figure 5:**
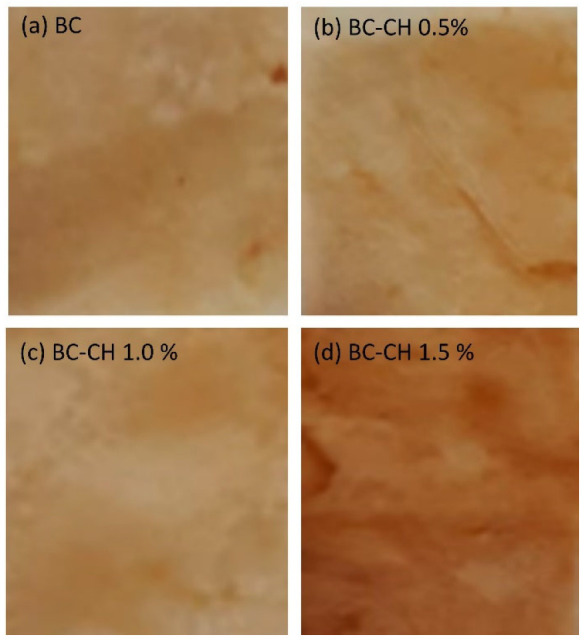
Color and opacity of: (**a**) BC, and (**b**–**d**) BC-CH films coated with various concentrations of chitosan.

**Figure 6:**
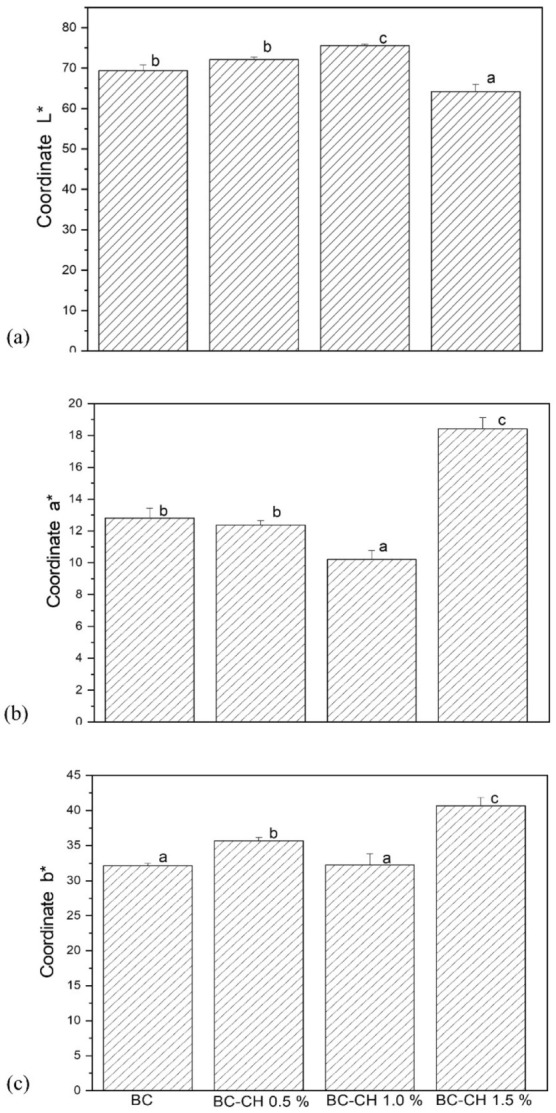
Parameters of colors: (**a**) L* (luminosity, 0 black to 100 white), (**b**) a* (−green to +red), and (**c**) b* (−blue to +yellow) of BC and BC-CH films coated with various concentrations of chitosan. Average ± standard deviation. Letters (a, b, c) indicate significant differences (*p* < 0.05) between treatments.

Regarding color analysis, a significant difference (*p* > 0.05) was found between treatments (Figure 5a–d). Although, in general, the films presented a brownish color, the L* values were similar for BC, BC-CH 0.5%, and BC-CH 1.0%, fluctuating between 69.38–75.54 while the lowest value was presented by BC-CH 1.5% (64.17 ± 1.82). These results agree with Ashraf et al. [19], who reported a reduction in L* values in chitosan/kombucha-tea films. Coordinate a* and b*, values ranged between 10.21–12.82 and 32.17–35.67 for BC, BC-CH 0.5%, and BC-CH 1.0%, whereas the values for BC-CH 1.5% were significantly higher (18.42 and 40.67, respectively; Figure 6). In general, positive values for the a* and b* parameters represent redness and yellowness, in agreement with that reported by Wang et al. [20] in chitosan/tea polyphenols films. In general, the results obtained in the color analysis of BC and BC-CH films may be associated with the components of the fermentation medium and cellular debris (nucleic acids, proteins, and bacteria, among others) incorporated into the BC polymeric matrix during the production process [16]. Additionally, it has also been reported that the incorporation of chitosan coatings can result in a loss of brightness and an increase in yellowness in the chitosan-coated cellulose-based paper [21], similar to that observed in films with a higher concentration of chitosan (BC-CH 1.5%).

**Figure 7:**
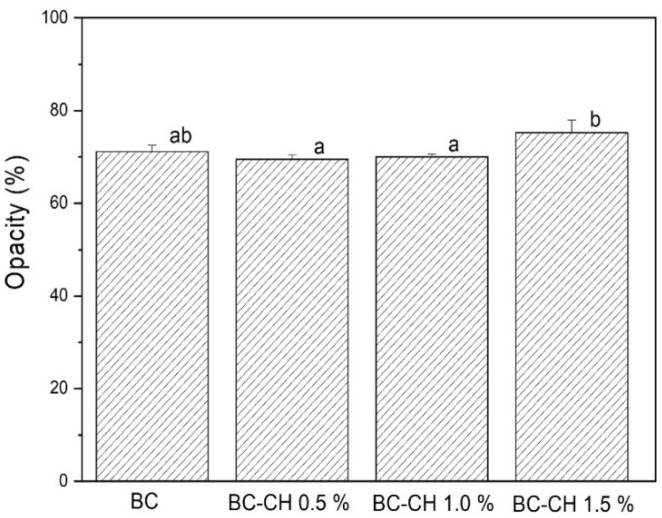
Opacity of BC and BC-CH films coated with various concentrations of chitosan. Average ± standard deviation. Letters (a, b) indicate significant differences (*p* < 0.05) between treatments.

On the other hand, regarding the opacity parameter, the highest value (*p* < 0.05) was presented by BC-CH 1.5% (75.24%), while the values found in BC (71.15%), BC-CH 0.5% (69.50%), and BC-CH 1.0% (70.03%) were very similar (*p* > 0.05) to each other (Figure 7), indicating that the film becomes opaquer as the concentration of chitosan in the coating increases. This result may be attributed to the fact that a high concentration of chitosan allows for a more closed polymer matrix with a lower depth of light penetration; a similar effect was reported by Fakhouri et al. [24] in films based on starch/gelatin containing high concentrations of starch.

#### 3.4.4. UV Visible Spectroscopy

**Figure 8:**
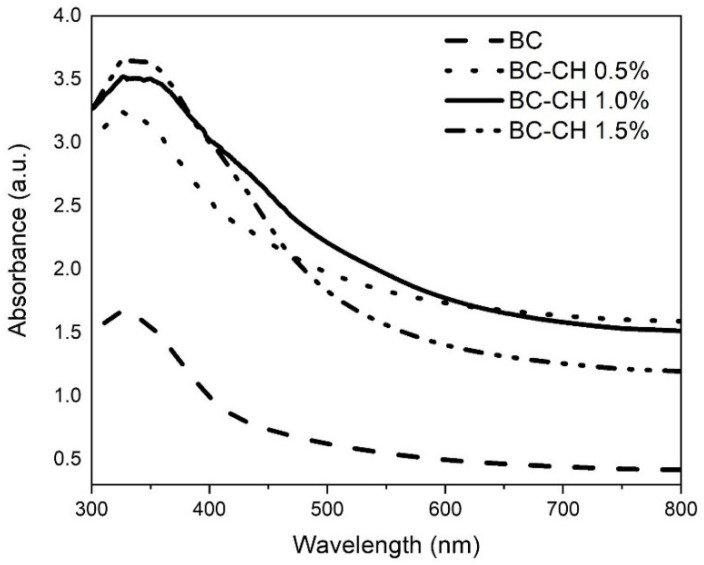
UV-Visible spectra of BC and BC-CH films coated with various concentrations of chitosan.

UV-Vis spectroscopy analyzed the optical properties of the BC and BC-CH films to determine their possible light shielding effect. Figure 8 shows a clear difference in absorbance between BC-CH and BC films. Particularly in the UV range (300–400 nm), BC-CH 1.5%, BC-CH 1.0%, and BC-CH 0.5% films showed a higher light absorption concerning BC films; the highest peak for all treatments was found at 327 nm (BC-CH= 3.24–3.65 a.u. and BC= 1.67 a.u.). Likewise, BC-CH films also showed higher light absorption than BC films in the VIS range (400–800 nm), with a decreased absorption spectrum until reaching a plateau at 800 nm. This barrier effect of films prepared in this research has also been reported in previous studies on chitosan/tea polyphenols [19], and cellulose-based films [22]. Additionally, as mentioned in the previous section, the higher light absorption found in BC-CH 1.5% can be attributed to the application of a coating with a higher concentration of chitosan, providing a more closed polymeric matrix that improves the light barrier properties [24].

#### 3.4.5. Solubility

**Figure 9:**
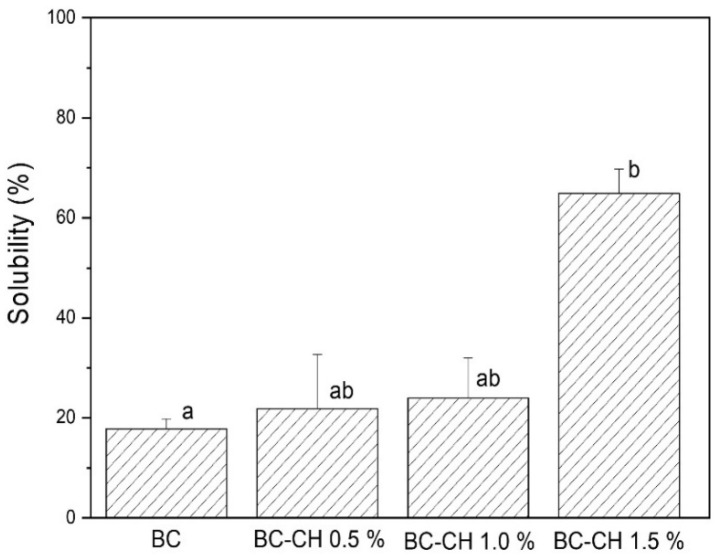
Solubility of BC and BC-CH films coated with various concentrations of chitosan. Average ± standard deviation. Letters (a, b) indicate significant differences (*p* < 0.05) between treatments.

As shown in Figure 9, applying the chitosan coating significantly influenced the solubility of BC and BC-CH films (*p* < 0.05). The lowest value was for BC (17.82%), followed by BC-CH 0.5% (21.82%) and BC-CH 1.0% (24.00%); the highest solubility value was presented by BC-CH 1.5% (64.91%). According to what was reported, cellulose is characterized as a water-insoluble polymer due to the formation of strong hydrogen bonds between molecules [41]. Therefore, the solubility values found in BC-CH films may be associated with the solubilization of the chitosan coating contained on the surface of the BC film due to the hydrophilic character of this polysaccharide, since values of up to 100% solubility in water (24 h at 25 °C) were reported in films made only with chitosan [42]. Additionally, the solubility values found in BC films could be related to the solubilization of some of the compounds (e.g., cellular debris, proteins, and polyphenols, among others) that remain incorporated in the BC matrix during its production process [16]. In addition, it was reported that the incorporation of tea polyphenols significantly increases film solubilization by up to 40% [20], in agreement with what was found in our research.

### 3.5. Biological Activities

#### 3.5.1. Antimicrobial Activity

Regarding the antimicrobial activity tests, it was observed that both BC and BC-CH films present antimicrobial activity against *S. aureus* and *E. coli* (Table 1). The activity values against *S. aureus* were significantly lower (*p* < 0.05) for BC films (5.27 ± 0.16 mm), whereas the values were very similar for BC-CH films (6.31–6.55 mm). In the case of *E. coli*, the antimicrobial activity values were highest for BC-CH 1.5% (8.25 ± 0.70 mm) followed by BC-CH 1.0% (7.89 ± 0.17 mm), BC-CH 0.5% (7.78 ± 0.23 mm), and BC (6.66 ± 0.72 mm). Similar antimicrobial activity results were reported in kombucha fermentation based on green tea, rosella flower, mango leather teas [43], and sugared snake fruit juices [44]. According to previous studies, the antimicrobial activity of BC films may be related to the content of organic acids. During kombucha fermentation: acetic, oxalic, L-lactic, citric, glucuronic, gluconic, and malic acids, ethanol, phenolic compounds, and flavonoids (e.g., catechins) are produced and incorporated into the polymeric matrix of the BC and were shown to inhibit a wide variety of gram-positive and gram-negative microorganisms [44,45,46]. Additionally, as can be seen in our results (Table 1), the application of chitosan coating increased (*p* < 0.05) the antimicrobial activity of the films. This effect can be attributed to the cationic nature of the polymer since the protonated amino group (NH_3_^+^) present at C-2 of this molecule can interact with the negatively charged microbial cell surface, resulting in the alteration of vital activities (e.g., alterations in cell surface morphology, changes in cell permeability, among others) in the microorganism [47]. Likewise, factors such as molecular weight, degree of deacetylation, concentration, and target organism, among others, can influence the antimicrobial effect of chitosan [19]. Therefore, compared to previous studies, our findings agree with those reported by Ashrafi et al. [19], who developed chitosan/kombucha-tea films effective against *E. coli* and *S. aureus.*

#### 3.5.2. Antioxidant Activity and Total Phenols Content

The values of DPPH, ABTS, and total phenolic content of BC and BC-CH films are shown in Table 2. In general, it can be observed that the antioxidant activity increased (*p* < 0.05) with the application of the chitosan coating in all treatments. The lowest values of antioxidant activity in DPPH and ABTS were for BC (13.56 ± 9.02% and 3.90 ± 1.10%, respectively), and the highest values were for BC-CH 1.0% (57.21 ± 2.04% and 24.57 ± 1.43%, respectively). According to what was reported in previous studies, the antioxidant activity of BC films may be associated with the presence of bioactive compounds such as phenolics, tannins, flavonoids, and catechins, among others, which are released and decomposed into their simpler forms during the kombucha fermentation process [44,48]. Likewise, our findings are also in agreement with those reported by Amorim et al. [16] in BC films produced in a green tea/black tea/glucose-based culture medium, whose antioxidant activity is related to the polyphenols released in the fermentation medium and the ability of these compounds to scavenge free radicals. Moreover, in agreement with our results, the antioxidant activity of films made from chitosan (7.5% radical scavenging activity) has also been demonstrated, whose effect is attributed to the reaction of the amino group residues (NH_3_^+^) of the C-2 with free radicals [49]. Similarly, our findings agree with Wang et al. [20] and Ashrafi et al. [19], who have evidenced the antioxidant activity of chitosan/tea polyphenols films and chitosan/kombucha-tea films, respectively. Additionally, the decrease in antioxidant activity in BC-CH 1.5% with respect to BC-CH 0.5% and BC-CH 1.0% could be associated with a partial encapsulation of the bioactive compounds in the polymeric matrix with the higher concentration of chitosan; a similar effect has been reported in films made with chitosan/*Rosmarinus officinalis* and chitosan/*Melaleuca alternifolia* [49].

Regarding total phenols content, the values of BC-CH films ranged from 2.14–2.16 mg GAE/g, and BC values were 0.64 ± 2.78 mg GAE/g. As mentioned above, phenolic compounds are high-level antioxidants characterized by their ability to scavenge free radicals. Therefore, the total phenols content in the films can be directly related to the content of polyphenols present in the kombucha culture medium, which increase during the fermentation process (from 3.55 mg/mL on day 1 to 6.55 mg/mL on day 12) due to the degradation of these complex compounds into simpler molecules by the effect of enzymes released by bacteria, yeasts, and fungi, as well as by the acidic environment of the medium [19]. In addition, Wang et al. [20] have reported the total phenol content in the ranges of 2.38–170.10 mg GAE/g in chitosan/tea polyphenols films whose values increase with increasing tea polyphenols content in the formulation.

## 4. Conclusions

This study successfully produced BC films in a green tea-based fermentation medium. Three CFSs of Newtonian character were developed with viscosities of 14–107 mPa.s, whose values increased with an increasing chitosan concentration. Three chitosan-coated film formulations (BC-CH 0.5%, BC-CH 1.0%, and BC-CH 1.5%) were developed with the BC films and CFS. The FTIR spectra of the films showed the prominent characteristic bands of cellulose and chitosan. The physicochemical characterization of the films showed that the incorporation of the chitosan coatings did not affect the thickness of the films; however, it did influence the color parameters (decreased brightness and increased redness and yellowness) and opacity (BC-CH 1.5%). Additionally, applying the chitosan coatings improved the optical properties of the films, such as light absorption in the UV-Vis range; moreover, the coatings’ application increased the films’ solubility by up to 64.91% (BC-CH 1.5%). Regarding the antimicrobial activity, incorporating chitosan coatings also improved the effectiveness of the films against *E. coli* and *S. aureus.* Likewise, BC-CH films presented higher values of antioxidant activity and total phenol content with respect to BC films. Finally, our results suggest that the BC-CH films developed in the present study show a potential application as an active packaging material in food. However, further studies on the physico-mechanical and gas barrier properties are needed to complement this research.

## Figures and Tables

**Table 1:** Antimicrobial activity of BC and BC-CH films coated with various concentrations of chitosan.

Films Samples	*Staphylococcus aureus* Inhibition Zone (mm)	*Escherichia coli* Inhibition Zone (mm)
BC	5.27 ± 0.16 ^a^	6.66 ± 0.72 ^a^
BC-CH 0.5%	6.35 ± 0.16 ^b^	7.78 ± 0.23 ^b^
BC-CH 1.0%	6.55 ± 0.06 ^c^	7.89 ± 0.17 ^b^
BC-CH 1.5%	6.31 ± 0.34 ^b^	8.25 ± 0.70 ^c^

Note: Average ± standard deviation. Letters (a, b, c) in columns indicate significant differences (*p* < 0.05) between treatments.

**Table 2:** Antioxidant activity and total phenolic content of BC and BC-CH films coated with various concentrations of chitosan.

Films Samples	DPPH Radical Scavenging Activity (%)	ABTS Radical Scavenging Activity (%)	Total Phenolic Content (mg GAE/g)
BC	13.56 ± 9.02 ^a^	3.90 ± 1.10 ^a^	0.64 ± 2.78 ^a^
BC-CH 0.5%	38.02 ± 7.5 ^c^	21.90 ± 1.43 ^c^	2.16 ± 0.15 ^b^
BC-CH 1.0%	57.71 ± 2.04 ^d^	24.57 ± 1.43 ^c^	2.45 ± 0.68 ^b^
BC-CH 1.5%	20.52 ± 0.22 ^b^	10.4 ± 0.90 ^b^	2.14 ± 0.68 ^b^

Note: Average ± standard deviation. Letters (a, b, c, d) in columns indicate significant differences (*p* < 0.05) between treatments.

## Data Availability

Not applicable.
